# Functional Carbazole–Cellulose Composite Binders for High-Stability Carbon Electrodes in Perovskite Solar Cells

**DOI:** 10.3390/nano15241868

**Published:** 2025-12-12

**Authors:** Fengming Guo, Junjie Wu, Yujing Li, Zilong Zhang, Maolin He, Lusheng Liang, Reza Keshavarzi, Peng Gao

**Affiliations:** 1 State Key Laboratory of Structural Chemistry, Fujian Institute of Research on the Structure of Matter, Chinese Academy of Sciences, Fuzhou 350002, China; fengming_g@163.com (F.G.); xmwujunjie@fjirsm.ac.cn (J.W.); xmliyujing@fjirsm.ac.cn (Y.L.); zhangzilong@fjirsm.ac.cn (Z.Z.); xmhemaolin@fjirsm.ac.cn (M.H.); lushengliang@fjirsm.ac.cn (L.L.); 2 Laboratory for Advanced Functional Materials, Xiamen Institute of Rare Earth Materials, Haixi Institute, Chinese Academy of Sciences, Xiamen 361021, China; 3 Fujian College, University of Chinese Academy of Sciences, Fuzhou 350002, China; 4 College of Chemistry, Fuzhou University, Fuzhou 350108, China; 5 Department of Chemistry, University of Isfahan, Isfahan 81746-73441, Iran

**Keywords:** carbon paste electrode, carbazole, cellulose, perovskite solar cells, device stability and efficiency

## Abstract

Perovskite solar cells (PSCs) based on metal halides have garnered significant attention due to their exceptional power conversion efficiency (PCE) and compatibility with low-temperature fabrication processes. However, the development of stable and inexpensive carbon electrodes remains hindered by issues such as insufficient conductivity at the carbon electrode/perovskite interface and weak coupling strength. In this study, we employed a functionalized carbazole–cellulose composite (C–Cz) as an alternative binder to construct highly stable carbon electrodes for PSCs. The incorporation of C–Cz enhances electron interactions through its conjugated carbazole moieties, while the cellulose backbone facilitates uniform dispersion of carbon particles and forms continuous transport pathways. These synergistic effects significantly optimize interfacial energy alignment and defect passivation. Ultimately, p-i-n PSCs fabricated with C–Cz carbon paste electrodes achieved a champion PCE of 16.79%, substantially outperforming the control device using a conventional PMMA binder (10.56%). Notably, the exceptional hydrophobicity and defect passivation capabilities of the C–Cz electrode substantially enhance device durability—maintaining over 95% of initial efficiency after 400 h of continuous maximum power point tracking irradiation. This study reveals an effective adhesive engineering strategy for robust, scalable carbon electrodes, paving new pathways for practical applications in stable perovskite photovoltaics.

## 1. Introduction

Carbon electrodes are attractive for perovskite solar cells (PSCs) due to their high chemical stability [1], good conductivity [2], and work function comparable to gold [3,4,5,6]. They can be deposited by scalable, low-temperature methods such as screen printing, doctor blading, inkjet printing, roll-to-roll transfer, and drop-casting [7,8,9,10,11,12,13]. Nevertheless, the application of commercial conductive carbon pastes in PSCs remains hindered by several critical drawbacks. First, their conductivity, typically derived from graphite, carbon black, or carbon nanotubes [14,15,16], is insufficient compared with noble-metal electrodes [17,18], increasing series resistance and thereby limiting the fill factor (FF) and power conversion efficiency (PCE) [19,20]. Second, poor interfacial contact between carbon electrodes and perovskite absorbers leads to high interfacial resistance and inefficient charge extraction [21,22,23,24]. Moreover, conventional pastes generally require high-temperature sintering (300–400 °C) to remove polymer binders and thickeners, a process that damages perovskite crystallinity or induces thermal decomposition [25]. In addition, certain organic solvents in these formulations may partially dissolve or degrade the perovskite layer during deposition, further compromising device stability [26,27].

**Figure 1:**
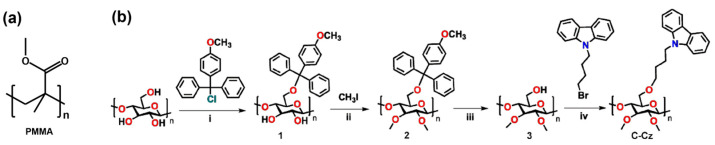
(**a**) PMMA structure diagram; (**b**) synthetic routes to C–Cz [37].

Polymethyl methacrylate (PMMA) has been widely used as a binder in carbon pastes due to its strong film-forming ability, mechanical robustness, and chemical stability [28] (Figure 1a). However, PMMA is electrically insulating and can form barriers between conductive particles, lowering conductivity and device efficiency [29,30]. Furthermore, as a synthetic polymer, PMMA is non-biodegradable and exhibits environmental persistence, which conflicts with the growing emphasis on sustainable electronic devices. Within the broader context of green and flexible electronics, researchers have explored alternative electrode composites such as the degradable elastomer Ecoflex or biodegradable polylactic acid (PLA) blended with carbon nanotubes (CNTs), sometimes incorporating liquid crystals like 5CB as templates to enhance conductivity and mechanical compliance [31,32,33]. Although these materials offer promising avenues for environmentally friendly devices, they often face challenges in achieving the high conductivity, energy level matching, and long-term operational stability required for efficient photovoltaic energy conversion. To address these limitations, natural and renewable cellulose derivatives have been explored as alternative binders [34,35]. Ethyl cellulose and hydroxypropyl cellulose, for instance, can improve film morphology and electrochemical properties, but they still require high-temperature sintering and contribute to insulating interfaces [25,36].

To overcome these bottlenecks, we designed and synthesized a carbazole-functionalized cellulose derivative (Figure 1b, Scheme S1), 6-O-[4-(9H-carbazol-9-yl)butyl]-2,3-di-O-methyl cellulose (C–Cz). Although it has been demonstrated in our previous work as an efficient bifunctional interlayer material capable of passivating interface defects and enhancing electron extraction [37]. This study innovatively applies it as a multifunctional binder in carbon-based electrodes and proposes a novel conductive polymer–carbon composite strategy. Specifically, conjugated carbazole groups were employed to construct hole-transport pathways between carbon particles, simultaneously fulfilling dual requirements for mechanical bonding and charge carrier transport, thereby significantly enhancing electrode conductivity. Concurrently, the cellulose framework ensured robust adhesion and, crucially, imparted excellent hydrophobicity to the electrode surface, forming an integrated moisture barrier. Finally, by introducing cellulose material C–Cz with carbazole functional groups into the carbon slurry, we successfully fabricated carbon electrodes with lower resistance and significantly improved interfacial contact. The PCE of p-i-n perovskite solar cells fabricated with this electrode increased from 10.56% to 16.79%. Moreover, after 400 h of continuous maximum power point tracking irradiation, the device maintained over 95% of its initial efficiency. This approach offers a novel technical solution for fabricating high-performance, stable, and cost-effective carbon electrodes.

## 2. Materials and Methods

Materials: All reagents and solvents used were commercially available without further purification. Formamidinium iodide (FAI), methylammonium bromide (MABr), and methylammonium chloride (MACl) were prepared following the synthetic protocols described in previous reports [38]. The other reagents used in this study included lead iodide (PbI_2_), lead bromide (PbBr_2_), and cesium iodide (99.9%), together with high-purity solvents such as anhydrous N,N-dimethylformamide (DMF, 99.8%) and dimethyl sulfoxide (DMSO, 99.9%), All purchased from TCI (Tokyo, Japan). Additional materials comprised chlorobenzene (CB,), bis(trifluoromethane)sulfonimide lithium salt (Li-TFSID, 99.95%, Sigma-Aldrich, St. Louis, MO, USA), 4-tert-butylpyridine (t-BP, 98%, TCI, Japan), 2,2′,7,7′-tetrakis[N,N-di(4-methoxyphenyl)amino]-9,9′-spirobifluorene (Spiro-OMeTAD, Derthon, Shenzhen, China), and poly(methyl methacrylate) (PMMA), highly conductive carbon black (HCCB, 99.99%, MTI), and chloroform (CF) All purchased from Sinopharm Group (Shanghai, China). 6-O-[4-(9H pyrazole-9-yl)butyl]-2,3-dioctyl cellulose (C–Cz) was synthesized according to the literature we previously reported (Scheme S1) [37].

Device Fabrication: preparation of substrate: Etched fluorine-doped tin oxide (FTO) glasses were cleaned with detergent, deionized water, acetone, and ethanol by sonicating for 15 min for each solvent. After drying in a drying cabinet at 70 °C for 2 h, FTO substrates were further cleaned with UV plasma for 15 min. Subsequently, a thin layer of SnO_2_ nanoparticle film (SnO_2_ colloid precursor diluted by deionized water, 1:3, weight ratio) was spin-coated on the FTO substrates at 3000 rpm for 30 s and annealed in ambient air at 150 °C for 30 min.

Deposition of the perovskite layer: First, prepare a solution of the (FAPbI_3_)_0.9_ (MAPbBr_3_)_0.1_ perovskite precursor. The initial precursor consists of PbI_2_, FAI, PbBr_2_, MACl, and MABr, dissolved in a mixed solvent of dimethylformamide (DMF) and dimethyl sulfoxide (DMSO) at a volume ratio of 9:1. After stirring the mixture for 30 min, add 57.5 μL of CsI solution (CsI was pre-dissolved in DMSO at a 1.5 M concentration to prepare a stock solution). Continue stirring under a nitrogen atmosphere for 24 h to obtain a uniform and stable perovskite precursor solution. At 8 s before the end of the program, add 200 μL of chlorobenzene onto the rotating substrate. Subsequently, heat-treat the FTO/SnO_2_/perovskite sample at 150 °C for 30 min. After cooling, the perovskite sample for the next step.

PSCP (conductive carbon paste using conventional PMMA as the binder)/CSCP (conductive carbon paste using C–Cz as the binder) was prepared as follows: Conductive Carbon Paste: Dissolve 15 mg of PMMA/ C–Cz in 1 mL of chloroform and stir for 3 h. Take the prepared Spiro-OMeTAD precursor solution (72.3 mg Spiro-OMeTAD in 1 mL chloroform, with additives including 17.5 µL Li-TFSI solution (520 mg/mL in acetonitrile) and 28.5 µL tBP). Mix the PMMA/ C–Cz solution with the Spiro-OMeTAD precursor solution at a 1:3 ratio, add 4 wt% highly conductive carbon black (HCCB), and stir for 24 h to achieve uniform dispersion, yielding the PSCP/CSCP conductive carbon paste. Despite extensive research on various alternative hole-transport materials (HTMs)—including cost-effective small molecules (e.g., azobenzotriazine), polymers, and inorganic compounds—aimed at enhancing stability and reducing costs [39,40], this study employed the widely adopted Spiro-OMeTAD as the standard hole-transport material. This ensures that the observed performance improvements are not confounded by variations in hole-transport layer (HTL) characteristics.

Preparation of Electrodes: Carbon electrodes were fabricated using the doctor-blade coating method. Perovskite-coated samples were fixed onto glass substrates, with 5 mm high-temperature-resistant tape applied along the cathode side and the edges sealed. The prepared devices were mounted on an automatic vacuum doctor-blade coater, and 300 µL of conductive carbon paste was dispensed at the blade’s starting position. PSCP and CSCP conductive carbon pastes were separately coated onto the perovskite layer at a speed of 15 mm s^−1^, producing carbon electrodes with an active area of 2 cm^2^. The devices were then annealed on a hot plate at 70 °C for 10 min. After annealing, the tape was removed, and the devices were cooled to obtain the complete solar cell modules.

Characterizations and Measurements: The water contact angle of the samples was determined using a DataPhysics contact angle analyzer (DataPhysics Instruments GmbH, Filderstadt, Germany). The surface and cross-sectional morphologies of the perovskite layers were examined by field-emission scanning electron microscopy (FE-SEM, Apreo S LoVac, Thermo, Waltham, MA, USA). Surface topography and roughness were further evaluated through atomic force microscopy (AFM, Oxford Jupiter XR, Oxford, UK). Steady-state photoluminescence (PL, excitation wavelength: 460 nm) and time-resolved photoluminescence (TRPL) spectra were acquired using an Edinburgh Instruments FLS 980 spectrometer (Edinburgh Instruments, Livingston, UK). It should be noted that due to the carbon electrode’s strong light absorption and scattering properties in the visible spectrum, it appears as a black opaque film at the macroscopic level. Since all PL/TRPL measurements in this study were performed from the FTO substrate side, they primarily reflect the radiative/non-radiative recombination behavior of the perovskite bulk phase, with weaker transient extraction responses at the carbon electrode interface. The photovoltaic characteristics were assessed under AM 1.5 G illumination (100 mW cm^−2^) with a solar simulator (Enli Tech, Kaohsiung City, Taiwan), while current density–voltage (J–V) curves were recorded using a Keithley 2401 source meter (Keithley Instruments, Solon, OH, USA) at a scan rate of 100 mV s^−1^. Measurements, including light-intensity-dependent VOC, dark current, transient photovoltage (TPV), transient photocurrent (TPC), and Mott–Schottky analyses, were performed in the dark at room temperature using an electrochemical workstation (Zennium Zahner, Kronach, Germany).

## 3. Results and Discussion

### 3.1. The Conductivity and Interface Morphology of C–Cz Carbon Electrodes

**Figure 2:**
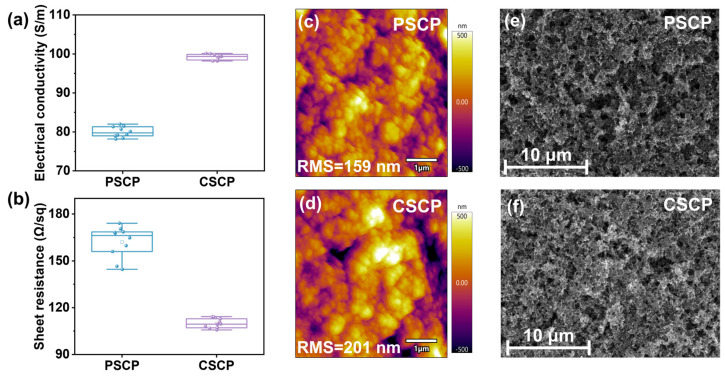
Boxplots of (**a**) conductivity and (**b**) sheet resistance of PSCP and CSCP conductive carbon pastes, AFM images of (**c**) PSCP and (**d**) CSCP conductive carbon pastes, and SEM top-view images of (**e**) PSCP and (**f**) CSCP.

The electrical conductivity and resistivity of the two carbon pastes were tested, with the results shown in Figure 2a,b and Table S1. Compared to PSCP, CSCP exhibits a higher conductivity of 100.13 S/m and a lower sheet resistance of 106.78 Ω/sq^−1^. This improvement is attributed to the presence of carbazole groups in the C–Cz used in CSCP, which exhibit strong π–π stacking interactions that facilitate charge transport between molecular chains [41]. In contrast, PMMA in PSCP is an insulating polymer that primarily serves as a matrix but performs poorly in charge conduction. Additionally, the cellulose in CSCP enables a more uniform dispersion of highly conductive carbon black, forming a continuous conductive network. On the other hand, due to the poor compatibility between PMMA and carbon black in PSCP, the conductive network is discontinuous or unevenly distributed, adversely affecting the overall conductivity.

The surface roughness obtained from AFM testing is shown in Figure 2c,d. The roughness of the PSCP electrode is 159 nm, while the roughness of the CSCP electrode is 201 nm. Comparing the conductivity and resistivity data from Figure 2a,b and Table S1, it is evident that although the roughness of the CSCP carbon film is higher than that of PSCP, its conductivity is better. This can be explained by the fact that for carbon materials, an increase in roughness typically indicates a thicker and more three-dimensional accumulation of conductive carbon material, enhancing the conductive paths in both vertical and horizontal directions [42]. Forming a three-dimensional carbon network effectively shortens the carrier transport path, reduces local series resistance, and enhances the effective conductivity of the composite electrode [43]. In CSCP, the cellulose modified with carbazole groups possesses flexible chain segments that assist in the uniform dispersion of carbon black particles, enabling closer packing and stronger interparticle adhesion. Although the surface becomes slightly rougher, the improved particle interconnection ensures a continuous conductive pathway with low sheet resistance. The improved electrical performance, therefore, originates from enhanced carbon-network connectivity and interfacial contact promoted by the C–Cz binder, rather than from the intrinsic conductivity of C–Cz itself.

In contrast, the PSCP with a PMMA matrix lacks π–π stacking interactions, leading to uneven distribution of carbon black particles and the formation of local insulating regions, thus increasing sheet resistance. However, the larger roughness in CSCP may increase contact resistance, affecting carrier transport efficiency. When in contact with the perovskite active layer, this could lead to interface defects or non-uniform electric field distribution, thereby increasing the hysteresis effect.

To investigate the surface and cross-sectional morphology of different carbon pastes, SEM analysis was performed, as shown in Figure 2e,f and Figure S1. From the SEM top-view and cross-sectional images, it is evident that the surface of the PSCP is relatively dense and smooth, with a more uniform distribution of particles and a tighter structure. In contrast, the surface of the CSCP shows larger and rougher particles, with significant porosity in some regions, resulting in a more loosely packed structure. This difference can be attributed to the fact that PMMA in PSCP, compared to C–Cz in CSCP, has superior film-forming capabilities, effectively encapsulating the carbon black particles and providing better adhesion, which helps to stabilize the particle distribution, leading to a denser and smoother carbon film. In CSCP, the carbazole groups and flexible chains of C–Cz promote the formation of a porous morphology during film deposition. This porous network facilitates the uniform dispersion and interconnection of carbon black particles, forming a more efficient conductive pathway throughout the electrode [44]. The open structure also enhances interfacial contact with the perovskite layer, which reduces interfacial recombination and suppresses ion accumulation at the interface rather than promoting ion migration. Overall, while the denser PSCP electrode provides higher mechanical strength, its limited interfacial contact restricts carrier extraction. In contrast, the porous and well-connected CSCP electrode enables more efficient hole transport and improved interfacial stability, thereby enhancing both the efficiency and operational durability of the device.

### 3.2. Interface and Charge Transport of PSCP and CSCP Carbon Electrodes

We also performed water contact angle testing, as shown in Figure S2. From the figure, it is evident that although PSCP (106.287°) is more hydrophobic than hydrophilic materials (>90°), it is less hydrophobic than CSCP (140.174°). The surface of PSCP may form a moisture-resistant barrier, but the risk of moisture ingress is relatively higher when exposed to humid environments for extended periods. In contrast, the hydrophobicity of CSCP is much higher (superhydrophobic surface), indicating extremely low surface energy, which effectively repels water and provides stronger protection to the perovskite layer. Its exceptional hydrophobic properties more effectively block environmental humidity, reducing the risk of perovskite degradation and thereby enhancing the long-term stability of the device. This is particularly important for unencapsulated devices. Although high hydrophobicity may affect interface adhesion, the carbazole groups in CSCP possess hole-transporting properties, which can optimize compatibility with spiro-OMeTAD and reduce interfacial resistance, thus balancing the demands of hydrophobicity and electrical performance. On the other hand, PMMA in PSCP, while stable as a traditional binder, lacks additional charge transport functionality [30].

**Figure 3:**
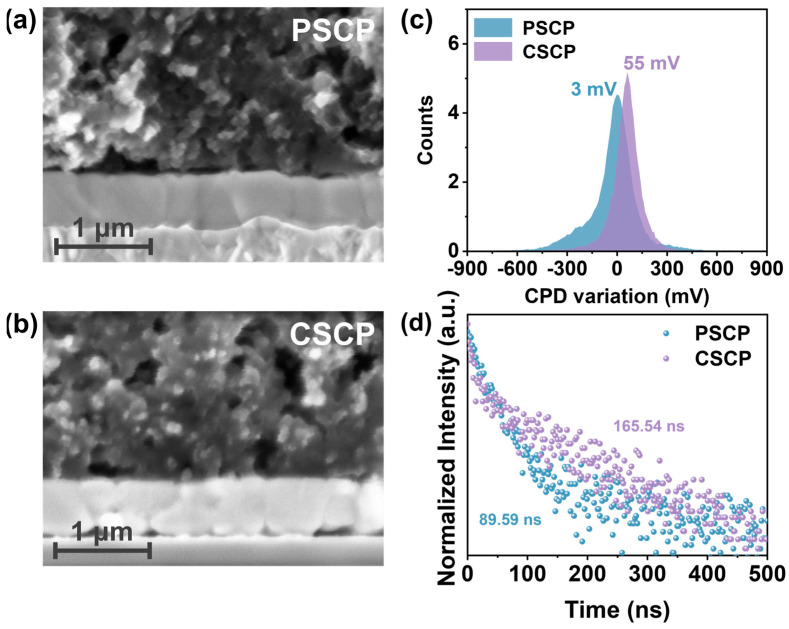
SEM cross-sectional images of (**a**) perovskite/PSCP and (**b**) perovskite/CSCP, (**c**) CPD variation of PSCP and CSCP carbon films, and (**d**) TRPL spectrum of the perovskite layer beneath different carbon electrodes (PSCP/CSCP).

Figure 3a,b present the cross-sectional structure of the perovskite and carbon electrode layers (PSCP and CSCP). The figure shows that the PSCP layer exhibits higher density, with more uniform particle packing and fewer voids. This is due to the compact structure caused by the PMMA component. Although the distribution of conductive carbon black is relatively uniform, it struggles to form a continuous conductive network, resulting in limited interfacial charge transport efficiency. Under prolonged operation, this may lead to accumulation effects, and the large gap between the PSCP layer and the perovskite layer leads to poor contact, which can facilitate charge recombination. In contrast, the CSCP layer has a relatively loose particle distribution and forms a porous network structure. Conductive carbon black within this porous structure creates continuous conductive pathways, improving conductivity and hole extraction efficiency.

Furthermore, the PVK/CSCP interface has better contact, which promotes charge transport and reduces charge recombination effects. While the PVK/PSCP interface is more compact, its poor conductivity and ion migration rate hinder efficient hole transport, limiting the overall device efficiency. The contact potential difference (CPD) of PSCP is 3 mV, while the CPD of CSCP is 55 mV (Figure 3c and Figure S3). This larger CPD value indicates that the CSCP electrode has a higher Fermi level [45], forming a more favorable energy level alignment with the perovskite layer, which helps to improve the V_OC_ of the device. Simultaneously, the elevated CPD value indicates that C–Cz not only alters the effective work function of the carbon electrode via its intrinsic HOMO energy level (~−5.48 eV) [37], but may also induce the formation of favorable interfacial dipoles and reduce interfacial recombination, thereby lowering the hole extraction barrier [46]. This mechanistic insight highlights the broader applicability of our strategy. The HOMO energy level of C–Cz analogues can be chemically tailored to match perovskite materials with different band structures [47].

To investigate the impact of different carbon paste electrodes on carrier extraction and transport, we performed photoluminescence (PL) spectroscopy measurements. As shown in Supplementary Figure S4, CSCP exhibits higher PL intensity, which can be attributed to the C–Cz -based carbon composite layer reducing trap density, thereby suppressing non-radiative recombination and enhancing radiative recombination efficiency. In contrast, stronger trap-assisted recombination in PSCP results in weaker PL. Furthermore, time-resolved photoluminescence (TRPL) was employed to quantify carrier dynamics, with curves fitted to a double-exponential decay function (Figure 3d), as summarized in Table S2. The fast lifetime component τ_1_ corresponds to interfacial carrier extraction and associated rapid recombination pathways, while the slow component τ_2_ primarily reflects recombination behavior within the perovskite bulk phase [48]. Devices based on CSCP exhibit significantly shorter τ_1_, indicating faster hole extraction at the interface. Concurrently, CSCP demonstrates longer τ_2_ and average lifetime τave, consistent with reduced deep-level traps and more complete bulk passivation. Thus, the synergistic effect of “shortened τ_1_ + increased τ_2_” results in enhanced PL intensity and longer TRPL carrier lifetime. These performance enhancements stem from the conjugated structure of the carbazole group inC–Cz, which exhibits strong charge transport properties and effectively suppresses carrier recombination.

### 3.3. Carrier Dynamics in PSCP and CSCP Devices

**Figure 4:**
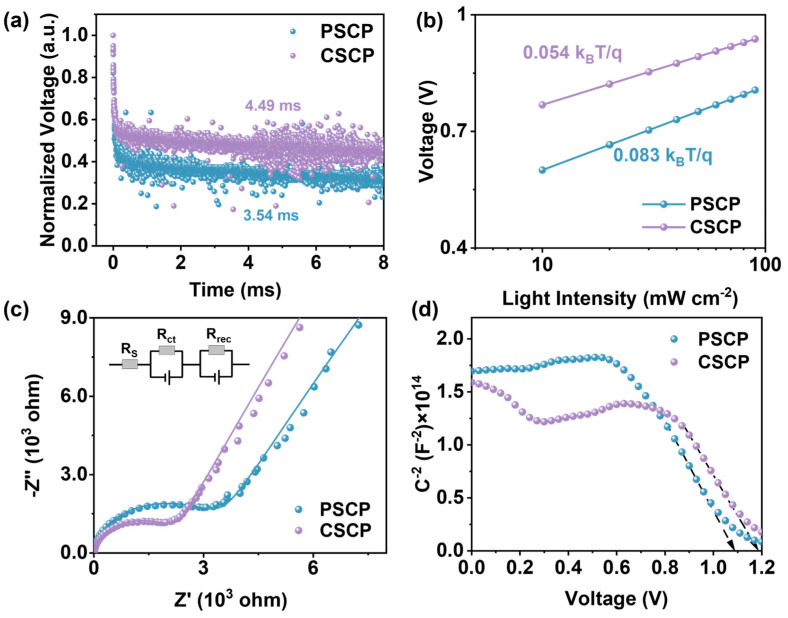
(**a**) TPV plots; (**b**) V_OC_ versus light intensity plots of PSCP, PSCs, and C-PSCs; (**c**) EIS plots of PSCP and CSCP-based PSCs, with the inset showing the equivalent circuit used for fitting the Nyquist plots; (**d**) Mott–Schottky plots.

The transient photovoltage (TPV) decay curves primarily reflect the carrier lifetime and the dynamics of their recombination. As shown in Figure 4a, the carrier lifetime for the CSCP-based device (4.49 ms) is longer than that for the PSCP-based device (3.54 ms), indicating a longer voltage retention time and slower recombination rate for CSCP. This suggests that CSCP effectively suppresses carrier recombination and reduces interfacial recombination. Incorporating carbazole groups in CSCP further enhances hole transport, improving V_OC_ and FF. In contrast, the faster decay observed in the PSCP-based device suggests the presence of a higher density of interfacial defects, leading to rapid charge recombination.

To further evaluate charge recombination characteristics, we examined the light intensity dependence of V_OC_ for both devices. As shown in Figure 4b, the PSCP-based device exhibits a higher ideality factor of 3.24, with a larger slope of 0.083 *k_B_T*/*q*, indicating significant non-radiative recombination, especially at interfaces and through defect states. This behavior is likely due to the poor dispersion of carbon black and the insulating nature of PMMA, leading to charge carrier accumulation. In contrast, the CSCP-based device shows a lower ideality factor of 2.10, closer to the typical range for high-quality perovskite solar cells (1~2), and a smaller slope of 0.054 *k_B_T*/*q*, suggesting that recombination is predominantly radiative with minimal non-radiative losses. These results demonstrate that incorporating carbazole groups in CSCP improves hole transport and minimizes interfacial defects, thus reducing charge recombination and enhancing device performance.

The electrochemical impedance spectroscopy (EIS) plots reveal the charge transfer resistance (R_ct_) and recombination resistance (R_rec_) in the device. By analyzing the data using an equivalent circuit model, the charge transport and recombination characteristics of the device can be understood. A higher Rct indicates low hole-transport efficiency at the electrode and perovskite interface. The value of Rrec is proportional to the diameter of the semicircle in the EIS plot, with a larger diameter indicating less charge recombination. The results of the EIS are shown in Figure 4c. The Rct values for PSCP and CSCP are 3.04 kΩ and 1.96 kΩ, respectively, while the Rrec values are 324 kΩ and 546 kΩ, respectively. This difference can be attributed to the higher density of interface defects in PSCP, which increases recombination rates. Additionally, PMMA’s insulating nature restricts charge transport. In contrast, the carbazole groups in C–Cz effectively reduce interface states and trap states, providing superior hole conduction pathways and enhancing hole extraction efficiency in CSCP.

The Mott–Schottky plot (Figure 4d) is typically used to measure the built-in potential (V_bi_) of PSCs. For the PSCP device, the V_bi_ is 1.08 V, which is lower than that of the CSCP device (1.18 V). The higher built-in potential of the CSCP device indicates a stronger built-in electric field, which more effectively drives the separation and transport of photogenerated charge carriers, thereby reducing recombination rates and resulting in a higher V_OC_. In contrast, the slightly lower built-in potential of the PSCP device corresponds to a weaker built-in electric field, leading to less efficient charge separation and a higher likelihood of recombination.

### 3.4. The Photovoltaic Performance of PSCP and CSCP Devices

**Figure 5:**
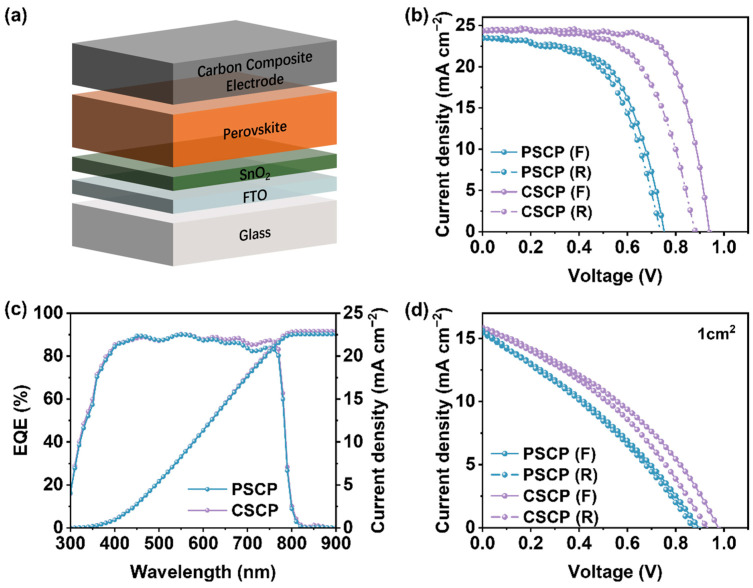
(**a**) Schematic diagram of PSCP/CSCP device structure. The carbon composite electrode consists of conductive carbon black, binder (PMMA orC–Cz), and Spiro-OMeTAD precursor uniformly dispersed in the carbon slurry. (**b**) Forward and reverse scan J-V curves of PSCP and CSCP devices with an active area of 0.1 cm^2^ under simulated AM 1.5G solar illumination (100 mW cm^−2^), (**c**) EQE spectra and integrated J_SC_ of the optimal device with an active area of 0.1 cm^2^, (**d**) J-V curves of PSCP and CSCP devices with an active area of 1 cm^2^.

To evaluate the performance of PSCP and CSCP in PSCs, we fabricated devices with the architecture FTO/SnO_2_(ETL)/Perovskite/Carbon Composite Electrode, where the carbon composite electrode was prepared by blade-coating the PSCP or CSCP carbon pastes containing conductive carbon black, polymer binder (PMMA orC–Cz), and an amount of Spiro-OMeTAD precursor, as illustrated in Figure 5a. Figure 5b presents the J-V curves of the optimal devices with an active area of 0.1 cm^2^ for both PSCP and CSCP devices. The photovoltaic parameters are summarized in Table S3. The optimal device with CSCP conductive carbon paste as the electrode achieved a power PCE of 16.79%, with V_OC_, FF, and J_SC_ values of 0.94 V, 72.81%, and 24.26 mA cm^−2^, respectively. Compared to the PSCP device, the CSCP carbon electrode contains C–Cz with strong π–π stacking interactions, which enhances the conductivity and lowers the resistivity of the carbon layer. Although CSCP exhibits higher conductivity and superior charge collection capability, its hysteresis index (HI) remains slightly higher than that of PSCP devices. This phenomenon may be attributed to the greater surface roughness of CSCP, which increases the contact resistance at the perovskite/CSCP interface. The resulting interface defects or localized electric field inhomogeneities readily promote ion migration from the perovskite layer to CSCP, thereby exacerbating the device’s hysteresis effect. On the other hand, the superior hydrophobicity of CSCP effectively suppresses moisture ingress, thereby slowing perovskite material degradation. Consequently, this device maintains superior stability compared to PSCP.

In contrast, PMMA in PSCP has insulating properties, resulting in higher resistance for the carbon paste. As a result, CSCP demonstrates superior performance in terms of photogenerated charge collection and transport, thereby improving device efficiency. Furthermore, the interface contact between the CSCP carbon electrode and the perovskite active layer is better, which helps reduce charge recombination and improves V_OC_ and J_SC_, leading to higher efficiency. We also tested the J-V curves of PSCP and CSCP devices with an active area of 1 cm^2^, as shown in Figure 5d, and the performance of the CSCP device remains superior to that of the PSCP device. The photovoltaic parameters are summarized in Table S4.

### 3.5. Performance and Stability Comparison of CSCP and PSCP Devices

**Figure 6:**
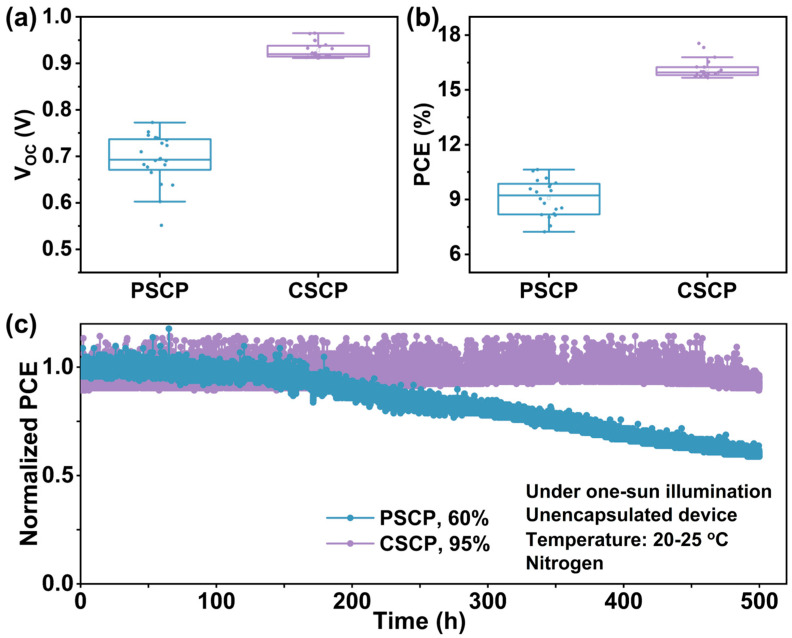
Boxplots of (**a**) V_OC_ and (**b**) PCE values extracted from the J-V measurements of PSCs based on PSCP and CSCP (20 independent devices). (**c**) Maximum power point tracking (MPPT) of the unencapsulated PSCP and CSCP PSCs comparing power output under 1 sun illumination, negative bias voltage, normal temperature, and N_2_ conditions.

Figure 6a,b and Figure S5 present the statistical distribution of various device parameters based on different carbon pastes. It can be seen that all parameters for the CSCP-based devices have improved. The PSCP devices exhibit the lowest V_OC_ and FF, which can be attributed to the insulating nature of PMMA in PSCP. Although PMMA serves as a binder and morphology controller in the carbon electrode, it significantly reduces the conductivity of the electrode [49], leading to higher charge transport resistance within the electrode in PSCP devices. This results in a higher series resistance, thereby reducing the device’s FF.

Additionally, due to its insulating properties, the energy level alignment between the carbon electrode and the perovskite layer may be less optimal, exacerbating the interface barrier effect and further lowering the V_OC_. In contrast, the C–Cz in CSCP exhibits strong π–π stacking interactions and excellent conductivity, effectively reducing series resistance and significantly enhancing charge collection efficiency and FF. Its superior electron transport properties and improved interfacial compatibility enhance the energy level alignment, resulting in a higher V_OC_. The integrated current density derived from the device’s external quantum efficiency (EQE) spectrum closely matches the value measured under simulated sunlight (within approximately a 5% range).

To further demonstrate the advantages of CSCP conductive carbon paste, we investigated the operational stability of the corresponding devices (Figure 5c and Figure S6). The results show that under the harsh conditions of unencapsulated devices subjected to continuous intense illumination (1 sun) and negative bias (PCSP: 0.53 V, CSCP: 0.73 V), long-term stability analysis of the maximum power point tracking (MPPT) output reveals significant advantages of CSCP as the electrode material for PSCs. The MPPT of the PSCP-based PSC rapidly decays (remaining at 60% of its initial value after 500 h), while the PSC with CSCP as the electrode retains more than 95% of its initial PCE after 400 h.

## 4. Conclusions

In summary, we propose a dual-function adhesive concept that simultaneously serves as both an electronic transport medium and an adhesive matrix. Specifically, C–Cz, acting as the adhesive, not only enhances the conductivity of carbon electrodes compared to conventional PMMA adhesives but also optimizes the energy level matching between the carbon layer and the perovskite. The conjugated carbazole groups within C–Cz promote π–π interactions with adjacent carbon particles and provide p-type charge transport pathways. The resulting porous carbon network establishes continuous conduction paths at the perovskite/electrode interface, effectively reducing series resistance, suppressing interfacial recombination, and accelerating hole extraction. Concurrently, the cellulose scaffold enhances carbon black adhesion, significantly improving device stability. Due to this synergistic effect, C–Cz-based PSCs achieve an optimal PCE of 16.79%, compared to 10.56% for the original device. More encouragingly, C–Cz-based devices exhibit outstanding stability under various environmental conditions. This is attributed to increased hydrophobicity, which effectively prevents moisture penetration into the perovskite film and mitigates perovskite degradation. Our work highlights the potential of multifunctional polymer binders in advancing carbon-based perovskite photovoltaic technology, contributing to the development of stable, low-cost, and high-efficiency carbon-based perovskite solar cells.

## Data Availability

The original contributions presented in this study are included in the article/Supplementary Materials. Further inquiries can be directed to the corresponding authors.

## Supplementary Materials

The following supporting information can be downloaded at: https://www.mdpi.com/article/10.3390/nano15241868/s1, Scheme S1: Synthetic routes toC–Cz. Reagents and conditions; Figure S1: SEM cross-sectional images (a,b) of PSCP and CSCP; Figure S2: Water contact angle of (a) PMCP and (b) CSCP; Figure S3: CPD distribution of (a) PSCP and (b) CSCP carbon films; Figure S4: PL spectrum of PSCP and CSCP; Figure S5: Distribution of (a) JSC and (b) extracted from J-V measurements of PSCP and CSCP based PSCs (20 independent devices); Figure S6: Long-term stability testing of unencapsulated PSCP and CSCP PSC devices under (a) 85 °C in an N_2_; environment and (b) air at 60 ± 10% relative humidity and 60 °C; Table S1: Conductivity and sheet resistance of PSCP and CSCP conductive carbon pastes; Table S2: TRPL fitting parameters of PSCP and CSCP based devices; Table S3: Photovoltaic parameters of optimal PSCs using PSCP and CSCP as electrodes. The values in parentheses represent the average and standard deviation measured over 20 devices; Table S4: Photovoltaic parameters of PSCP and CSCP devices with an active area of 1 cm^2^; Table S5: Comparison of commonly used binders in carbon pastes for carbon-based perovskite solar cells.

## References

[1] Rezakhani S., Shahroosvand H., Gao P., Nazeeruddin M.K. (2025). Innovating Carbon-Based Perovskite Solar Cells: The Role of a CN-Anchoring Self-Assembled Molecular Layer in Efficiency and Stability. J. Mater. Chem. A.

[2] Liang L., Cai Y., Li X., Nazeeruddin M.K., Gao P. (2018). All That Glitters Is Not Gold: Recent Progress of Alternative Counter Electrodes for Perovskite Solar Cells. Nano Energy.

[3] Ferguson V., Silva S.R.P., Zhang W. (2019). Carbon Materials in Perovskite Solar Cells: Prospects and Future Challenges. Energy Environ. Mater..

[4] Hadadian M., Smått J.-H., Correa-Baena J.-P. (2020). The Role of Carbon-Based Materials in Enhancing the Stability of Perovskite Solar Cells. Energy Environ. Sci..

[5] Que M., Zhang B., Chen J., Yin X., Yun S. (2021). Carbon-Based Electrodes for Perovskite Solar Cells. Mater. Adv..

[6] Omrani M., Keshavarzi R., Abdi-Jalebi M., Gao P. (2022). Impacts of Plasmonic Nanoparticles Incorporation and Interface Energy Alignment for Highly Efficient Carbon-Based Perovskite Solar Cells. Sci. Rep..

[7] Dubey R., Guruviah V. (2019). Review of Carbon-Based Electrode Materials for Supercapacitor Energy Storage. Ionics.

[8] Chen H., Yang S. (2019). Methods and Strategies for Achieving High-Performance Carbon-Based Perovskite Solar Cells without Hole Transport Materials. J. Mater. Chem. A.

[9] Babu V., Fuentes Pineda R., Ahmad T., Alvarez A.O., Castriotta L.A., Di Carlo A., Fabregat-Santiago F., Wojciechowski K. (2020). Improved Stability of Inverted and Flexible Perovskite Solar Cells with Carbon Electrode. ACS Appl. Energy Mater..

[10] Gan Y., Sun J., Guo P., Jiang H., Li J., Zhu H., Fan X., Huang L., Wang Y. (2023). Advances in the Research of Carbon Electrodes for Perovskite Solar Cells. Dalton Trans..

[11] Pradid P., Sanglee K., Thongprong N., Chuangchote S. (2021). Carbon Electrodes in Perovskite Photovoltaics. Materials.

[12] Liang L., Cai Y., Gao P. (2022). A Facile Gas-Driven Ink Spray (GDIS) Deposition Strategy toward Hole-Conductor-Free Carbon-Based Perovskite Solar Cells. Emergent Mater..

[13] Cai Y., Liang L., Gao P. (2018). Promise of Commercialization: Carbon Materials for Low-Cost Perovskite Solar Cells. Chin. Phys. B.

[14] Wu M., Sun M., Zhou H., Ma J., Ma T. (2020). Carbon Counter Electrodes in Dye-Sensitized and Perovskite Solar Cells. Adv. Funct. Mater..

[15] Forouzandeh M., Heidariramsheh M., Heydarnezhad H.R., Nikbakht H., Stefanelli M., Vesce L., Taghavinia N. (2024). Enhanced Carbon-Based Back Contact Electrodes for Perovskite Solar Cells: Effect of Carbon Paste Composition on Performance and Stability. Carbon.

[16] Pandey S., Karakoti M., Bhardwaj D., Tatrari G., Sharma R., Pandey L., Lee M.-J., Sahoo N.G. (2023). Recent Advances in Carbon-Based Materials for High-Performance Perovskite Solar Cells: Gaps, Challenges and Fulfillment. Nanoscale Adv..

[17] Lalpour N., Mirkhani V., Keshavarzi R., Moghadam M., Tangestaninejad S., Mohammadpoor-Baltork I., Gao P. (2023). Self-Healing Perovskite Solar Cells Based on Copolymer-Templated TiO2 Electron Transport Layer. Sci. Rep..

[18] Don M.F., Ekanayake P., Jennings J.R., Nakajima H., Lim C.M. (2022). Graphite/Carbon Black Counter Electrode Deposition Methods to Improve the Efficiency and Stability of Hole-Transport-Layer-Free Perovskite Solar Cells. ACS Omega.

[19] He J., Bai Y., Luo Z., Ran R., Zhou W., Wang W., Shao Z. (2025). Advanced Carbon-Based Rear Electrodes for Low-Cost and Efficient Perovskite Solar Cells. Energy Environ. Sci..

[20] Chen R., Zhang W., Guan X., Raza H., Zhang S., Zhang Y., Troshin P.A., Kuklin S.A., Liu Z., Chen W. (2022). Rear Electrode Materials for Perovskite Solar Cells. Adv. Funct. Mater..

[21] Aftabuzzaman M., Lu C., Kim H.K. (2020). Recent Progress on Nanostructured Carbon-Based Counter/Back Electrodes for High-Performance Dye-Sensitized and Perovskite Solar Cells. Nanoscale.

[22] Meng X., Zhou J., Hou J., Tao X., Cheung S.H., So S.K., Yang S. (2018). Versatility of Carbon Enables All Carbon Based Perovskite Solar Cells to Achieve High Efficiency and High Stability. Adv. Mater..

[23] Shao S., Loi M.A. (2020). The Role of the Interfaces in Perovskite Solar Cells. Adv. Mater. Inter..

[24] Xie H., Lei J., Zhu Z., Xu X., Li D., Xu J., Pan Y., Yin X. (2025). Practical Interface Engineering between Perovskite and Carbon Electrode in Regular Carbon-Based Perovskite Solar Cells. ACS Appl. Mater. Interfaces.

[25] Tsuji R., Tanaka K., Oishi K., Shioki T., Satone H., Ito S. (2023). Role and Function of Polymer Binder Thickeners in Carbon Pastes for Multiporous-Layered-Electrode Perovskite Solar Cells. Chem. Mater..

[26] Boyd C.C., Cheacharoen R., Leijtens T., McGehee M.D. (2019). Understanding Degradation Mechanisms and Improving Stability of Perovskite Photovoltaics. Chem. Rev..

[27] Miah M.H., Rahman M.B., Nur-E-Alam M., Islam M.A., Shahinuzzaman M., Rahman M.R., Ullah M.H., Khandaker M.U. (2025). Key Degradation Mechanisms of Perovskite Solar Cells and Strategies for Enhanced Stability: Issues and Prospects. RSC Adv..

[28] Wang A., Xu H., Zhou Q., Liu X., Li Z., Gao R., Wu N., Guo Y., Li H., Zhang L. (2016). A New All-Solid-State Hyperbranched Star Polymer Electrolyte for Lithium Ion Batteries: Synthesis and Electrochemical Properties. Electrochim. Acta.

[29] Shaari H.A.H., Ramli M.M., Mohtar M.N., Rahman N.A., Ahmad A. (2021). Synthesis and Conductivity Studies of Poly (Methyl Methacrylate) (PMMA) by Co-Polymerization and Blending with Polyaniline (PANi). Polymers.

[30] Ali U., Karim K.J.B.A., Buang N.A. (2015). A Review of the Properties and Applications of Poly (Methyl Methacrylate) (PMMA). Polym. Rev..

[31] Lv K., Tian G., Yan Y., Zhou H., Fan Q., Liang L., Liu N., Wang D., Song Z., Xu F. (2024). Stretchable Carbon Nanotube/Ecoflex Conductive Elastomer Films toward Multifunctional Wearable Electronics. Chem. Eng. J..

[32] Shi S., Chen Y., Jing J., Yang L. (2019). Preparation and 3D-Printing of Highly Conductive Polylactic Acid/Carbon Nanotube Nanocomposites via Local Enrichment Strategy. RSC Adv..

[33] Liu Y., Chang C., Ye W., Guo X. (2023). Composite of DNA-Stabilized Multiwalled Carbon Nanotube and Nematic Liquid Crystal for Display Performance Optimization. ACS Appl. Electron. Mater..

[34] Mili M., Hashmi S.A.R., Ather M., Hada V., Markandeya N., Kamble S., Mohapatra M., Rathore S.K.S., Srivastava A.K., Verma S. (2022). Novel Lignin as Natural-Biodegradable Binder for Various Sectors—A Review. J. Appl. Polym. Sci..

[35] Shaghaleh H., Xu X., Wang S. (2018). Current Progress in Production of Biopolymeric Materials Based on Cellulose, Cellulose Nanofibers, and Cellulose Derivatives. RSC Adv..

[36] Chasta G., Bhakar U., Suthar D., Lamba R., Dhaka M.S. (2023). Impact of Ethyl Cellulose Variation on Microstructural and Electrochemical Properties of Spin Coated YSZ Electrolyte Thin Films for SOFCs: Slurry Composition Evolution. Ceram. Int..

[37] Zhang Z., Wang C., Li F., Liang L., Huang L., Chen L., Ni Y., Gao P., Wu H. (2023). Bifunctional Cellulose Interlayer Enabled Efficient Perovskite Solar Cells with Simultaneously Enhanced Efficiency and Stability. Adv. Sci..

[38] Son D.-Y., Kim S.-G., Seo J.-Y., Lee S.-H., Shin H., Lee D., Park N.-G. (2018). Universal Approach toward Hysteresis−Free Perovskite Solar Cell via Defect Engineering. J. Am. Chem. Soc..

[39] Salunke J., Guo X., Liu M., Lin Z., Candeias N.R., Priimagi A., Chang J., Vivo P. (2020). N-Substituted Phenothiazines as Environmentally Friendly Hole-Transporting Materials for Low-Cost and Highly Stable Halide Perovskite Solar Cells. ACS Omega.

[40] Magomedov A., Paek S., Gratia P., Kasparavicius E., Daskeviciene M., Kamarauskas E., Gruodis A., Jankauskas V., Kantminiene K., Cho K.T. (2018). Diphenylamine-Substituted Carbazole-Based Hole Transporting Materials for Perovskite Solar Cells: Influence of Isomeric Derivatives. Adv. Funct. Mater..

[41] Hlel A., Mabrouk A., Chemek M., Khalifa I.B., Alimi K. (2015). A DFT Study of Charge-Transfer and Opto-Electronic Properties of Some New Materials Involving Carbazole Units. Comput. Condens. Matter.

[42] Zhang Z., Yang R., Wang Y., Xu K., Dai W., Zhang J., Li M., Li L., Guo Y., Qin Y. (2024). Enhanced Thermal Conductivity and Reduced Thermal Resistance in Carbon Fiber-Based Thermal Interface Materials with Vertically Aligned Structure. J. Mater. Chem. A.

[43] Zhao H.-Y., Yu M.-Y., Liu J., Li X., Min P., Yu Z.-Z. (2022). Efficient Preconstruction of Three-Dimensional Graphene Networks for Thermally Conductive Polymer Composites. Nano-Micro Lett..

[44] Hou M., Pan Y., He F., Xu K., Zhang H., Zhou Y., Zhao B., Chen Y., Liu M. (2022). Manipulating and Optimizing the Hierarchically Porous Electrode Structures for Rapid Mass Transport in Solid Oxide Cells. Adv. Funct. Mater..

[45] Rajendran M.V., Alagumalai A., Ganesan S., Menon V.S., Raman R.K., Thangavelu S.A.G., Krishnamoorthy A. (2023). Design and Synthesis of Multifaceted Dicyanomethylene Rhodanine Linked Thiophene: A SnO x–Perovskite Dual Interface Modifier Facilitating Enhanced Device Performance through Improved Fermi Level Alignment, Defect Passivation and Reduced Energy Loss. Sustain. Energy Fuels.

[46] Mavlonov A., Negami T., Kawano Y., Kojima K., Kitaguchi K., Azuma J., Minemoto T. (2024). Influence of Valence Band Offset at the Hole Transport Material/CH3NH3PbI3 Interface on Device Performance Using Fluorinated Spiro-OMeTAD. ACS Appl. Energy Mater..

[47] Saha N., Brunetti G., Carlo A.D., Ciminelli C. (2025). Efficiency Boost of Perovskite Solar Cell in Homojunction Configuration through Tailored Band Alignment and p–n Doping Profile. J. Phys. Energy.

[48] Wu J., He M., Liu C., Gao P. (2025). Charge Dynamics and Defect States under “Spot-Light”: Spectroscopic Insights into Halide Perovskite Solar Cells. Adv. Photonics Res..

[49] Nguyen V.A., Kuss C. (2020). Conducting Polymer-Based Binders for Lithium-Ion Batteries and Beyond. J. Electrochem. Soc..

