# Supplementary material for: Functional Carbazole–Cellulose Composite Binders for High-Stability Carbon Electrodes in Perovskite Solar Cells
Source: Nanomaterials (Basel). 2025 Dec 12;15(24):1868. doi: 10.3390/nano15241868 (PMC12735854; doi:10.3390/nano15241868)
Supplement: Supplementary file 1 [file nanomaterials-15-01868-s001.zip › nanomaterials-4031534-supplementary.pdf]

Supporting Information

Functional Carbazole–Cellulose Composite Binders for High-Stability Carbon Electrodes in Perovskite Solar Cells

Fengming Guo [1,2,3,†], Junjie Wu [1,2,3,4,†], Yujing Li [1,2,3], Zilong Zhang [1,2,3], Maolin He [1,2,3,4], Lusheng Liang [1,2,3], Reza Keshavarzi [5,*] and Peng Gao [1,2,3,4,*]

[1] State Key Laboratory of Structural Chemistry, Fujian Institute of Research on the Structure of Matter, Chinese Academy of Sciences, Fuzhou 350002, China; fengming_g@163.com (F.G.); xmwujunjie@fjirsm.ac.cn (J.W.); xmliyujing@fjirsm.ac.cn (Y.L.); zhangzilong@fjirsm.ac.cn (Z.Z.); xmhemaolin@fjirsm.ac.cn (M.H.); lushengliang@fjirsm.ac.cn (L.L.)

[2] Laboratory for Advanced Functional Materials, Xiamen Institute of Rare Earth Materials, Haixi Institute, Chinese Academy of Sciences, Xiamen 361021, China

[3] Fujian College, University of Chinese Academy of Sciences, Fuzhou 350002, China

[4] College of Chemistry, Fuzhou University, Fuzhou 350108, China

[5] Department of Chemistry, University of Isfahan, Isfahan 81746-73441, Iran

* Correspondence: r.keshavarzi85@gmail.com (R.K.); peng.gao@fjirsm.ac.cn (P.G.)

† These authors contributed equally to this work.

Supporting Information

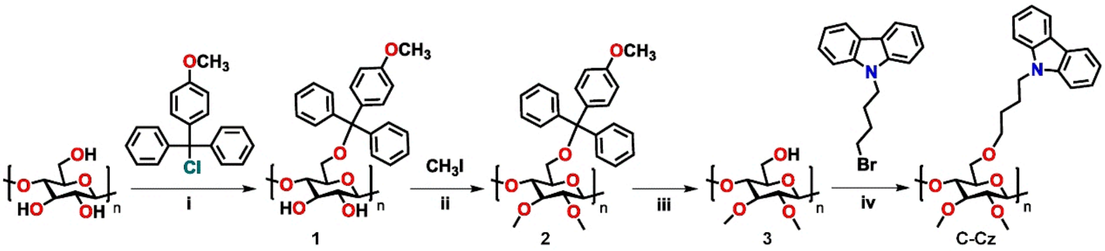

Scheme S1. Synthetic routes to C-Cz. Reagents and conditions: (i) DMAc:LiCl, 4-methoxytriphenylmethyl chloride, pyridine, 80 °C, 8 h; (ii) CH_3I, NaH, DMSO, 70 °C, 24 h; 2 (iii) THF, concentrated HCl, rt, 24 h; (iv) 9-(4-bromobutyl)-9H-carbazole, DMF, NaH, tetran-butylammonium iodide, 60 °C, 48 h.

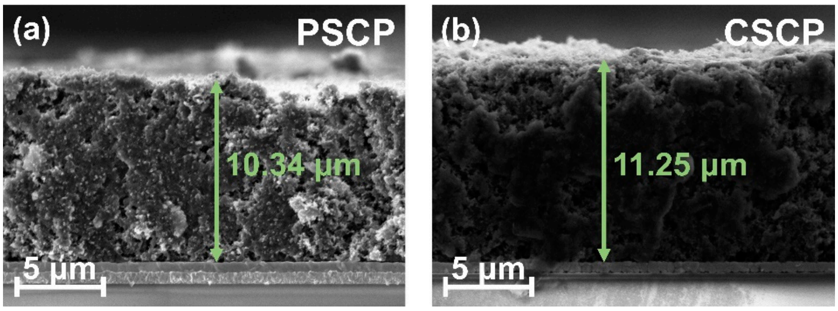

Figure S1. SEM cross-sectional images (a, b) of PSCP and CSCP.

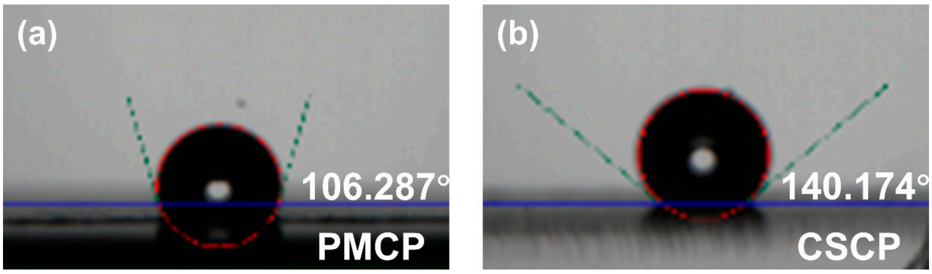

Figure S2. Water contact angle of (a) PMCP and (b) CSCP.

Supporting Information

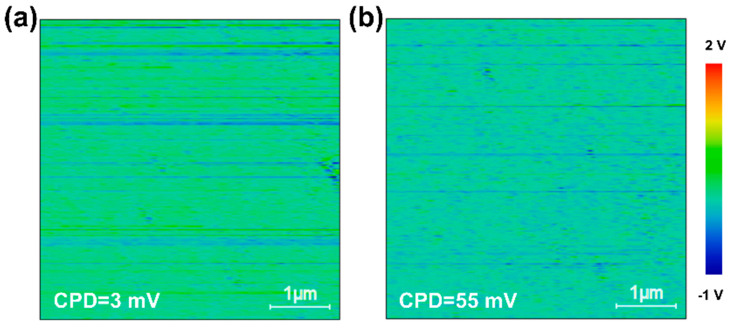

Figure S3. CPD distribution of (a) PSCP and (b) CSCP carbon films.

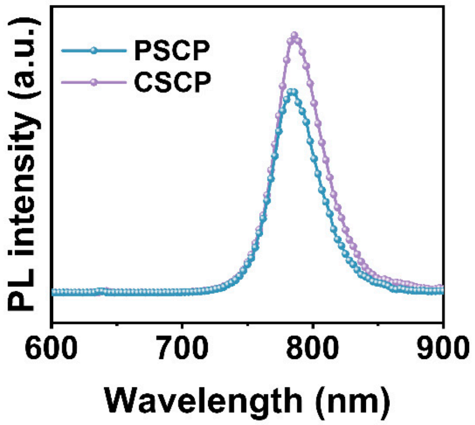

Figure S4. PL spectrum of PSCP and CSCP.

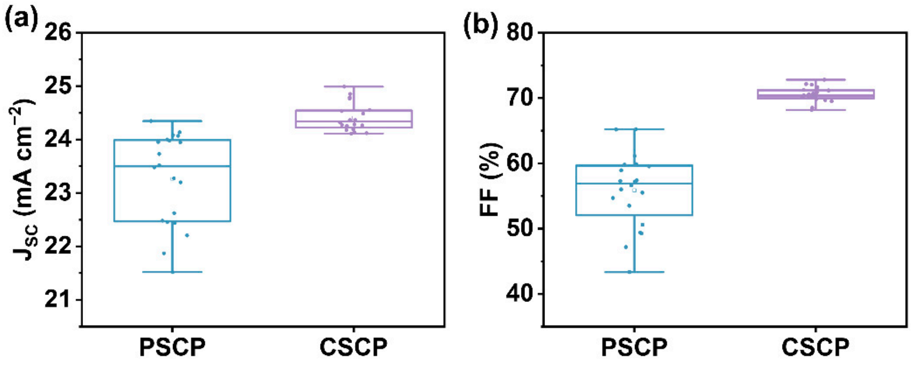

Figure S5. Distribution of (a) J_{SC} and (b) extracted from *J-V* measurements of PSCP and CSCP based PSCs (20 independent devices).

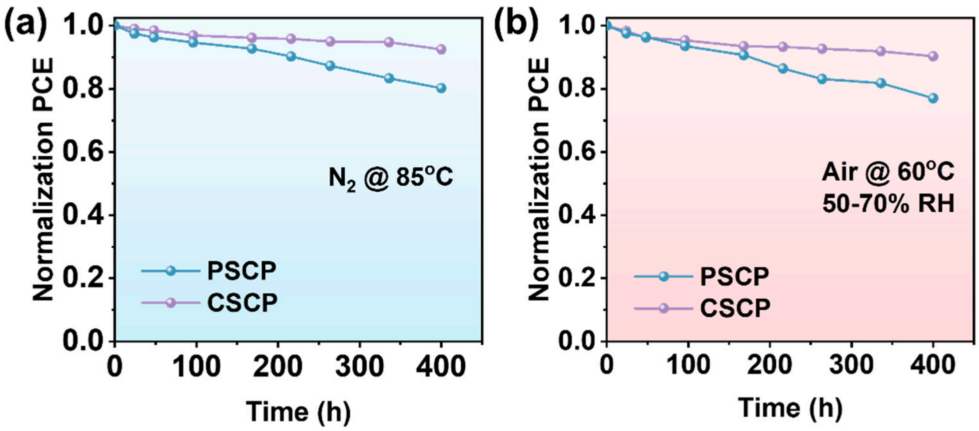

Figure S6. Long-term stability testing of unencapsulated PSCP and CSCP PSC devices under (a) 85°C in an N_2 environment and (b) air at 60 ± 10% relative humidity and 60°C.

Table S1. Conductivity and sheet resistance of PSCP and CSCP conductive carbon pastes.

Sample	Conductivity (S/m)	Resistance (Ω/sq)
PSCP	82.01	167.66
CSCP	100.13	106.78

Table S2. TRPL fitting parameters of PSCP and CSCP based devices.

Sample	A_1	τ_1 (ns)	A_2	τ_2 (ns)	τ_{AVE} (ns)
PSCP	0.70	28.62	0.18	139.51	89.59
CSCP	0.51	8.86	0.36	176.71	165.54

Supporting Information

Table S3. Photovoltaic parameters of optimal PSCs using PSCP and CSCP as electrodes. The values in parentheses represent the average and standard deviation measured over 20 devices.

Devices	Area (cm^2)	V_{OC} (V)	PCE (%)	*FF* (%)	J_{SC} (mA cm^{-2})
PSCP	0.1	0.75 (0.69 ± 0.055)	10.63 (9.07 ± 0.998)	58.94 (55.89 ± 5.695)	23.52 (23.26 ± 0.860)
CSCP	0.1	0.94 (0.92 ± 0.014)	16.79 (15.96 ± 0.307)	72.81 (70.32 ± 1.015)	24.26 (24.35 ± 0.204)

Table S4. Photovoltaic parameters of PSCP and CSCP devices with an active area of 1 cm^2.

Devices	Scan direction	V_{OC} (V)	PCE (%)	*FF* (%)	J_{SC} (mA cm^{-2})
1cm^2 PSCP	Forward	0.90	4.41	31.50	15.43
	Reverse	0.87	4.29	30.98	15.64
1cm^2 CSCP	Forward	0.98	5.71	36.39	15.86
	Reverse	0.93	5.29	35.28	15.87

Table S5. Comparison of commonly used binders in carbon pastes for carbon-based perovskite solar cells.

Binder	Electrical Properties	Technology	Interfacial Properties	Stability	Environmental Aspect
Ethyl Cellulose (EC)	Insulating	Requires high-temperature sintering	Good dispersion, but high resistance	Moderate; moisture-sensitive	Renewable biodegradable
Polymethyl Methacrylate (PMMA)	Highly insulating	Low-temperature solution processing	Causes insulating gaps between carbon particles	Moderate	Non-degradable
Hydroxypropyl Cellulose (HPC)	Insulating	Medium-temperature drying	TInterface charge transport is restricted	Moderate	Renewable
Poly(vinylidene fluoride) (PVDF)	Insulating	Low-temperature processing	High density, but energy levels do not match	Moderate; moisture-sensitive	Non-degradable
This work (C-Cz)	noninsulated	Low-temperature processable No sintering required	More conductive pathways, excellent dispersion, reduced porosity	High stability due to hydrophobicity and cellulose backbone	Renewable biodegradable environmentally friendly
